# Exploring Electrophysiological Responses to Hypnosis in Patients with Fibromyalgia

**DOI:** 10.3390/brainsci14111047

**Published:** 2024-10-23

**Authors:** Pradeep Kumar Govindaiah, A. Adarsh, Rajanikant Panda, Olivia Gosseries, Nicole Malaise, Irène Salamun, Luaba Tshibanda, Steven Laureys, Vincent Bonhomme, Marie-Elisabeth Faymonville, Audrey Vanhaudenhuyse, Aminata Bicego

**Affiliations:** 1 Centre for Brain Research, Indian Institute of Science Campus, Bengaluru 560012, India; pradeepkg@alum.iisc.ac.in; 2 MILE Lab, Department of Electrical Engineering, Indian Institute of Science, Bengaluru 560012, India; adarsha02@gmail.com; 3 Department of Neurology, University of California San Francisco (UCSF), San Francisco, CA 94158, USA; bk.bme.rajanikant@gmail.com; 4 Coma Science Group, GIGA Consciousness, Liege University, 4000 Liege, Belgium; ogosseries@uliege.be; 5 Centre du Cerveau2, Liege University Hospital, 4000 Liege, Belgium; 6 Conscious Care Lab, GIGA Consciousness, Liege University, 4000 Liege, Belgium; mfaymonville@chuliege.be; 7 Interdisciplinary Algology Center, Liege University Hospital, 4000 Liege, Belgium; nicole.malaise@chuliege.be (N.M.); isalamun@chuliege.be (I.S.); vincent.bonhomme@chuliege.be (V.B.); 8 Department of Radiology, Liege University Hospital, 4000 Liege, Belgium; l.tshibanda@chuliege.be; 9 CERVO Brain Research Centre, Laval University, Québec, QC G1V 0A6, Canada; steven.laureys@uliege.be; 10 Anesthesia, Critical Care and Pain Medicine Research, Harvard Medical School, Beth Israel Deaconess Medical Center, Boston, MA 02215, USA; 11 Consciousness Science Institute, Hangzhou Normal University, Hangzhou 310030, China; 12 Anesthesia and Perioperative Neuroscience Laboratory, GIGA Consciousness, Liege University, 4000 Liege, Belgium; 13 Department of Anesthesia and Intensive Care Medicine, Liege University Hospital, 4000 Liege, Belgium; 14 Oncology Integrated Arsen Bury Center, University Hospital of Liege, 4000 Liege, Belgium

**Keywords:** hypnosis, fibromyalgia, electroencephalography, neural oscillations

## Abstract

**Background/Objectives:** Hypnosis shows great potential for managing patients suffering from fibromyalgia and chronic pain. Several studies have highlighted its efficacy in improving pain, quality of life, and reducing psychological distress. Despite its known feasibility and efficacy, the mechanisms of action remain poorly understood. Building on these insights, this innovative study aims to assess neural activity during hypnosis in fibromyalgia patients using high-density electroencephalography (EEG) and self-reported measures. **Methods**: Thirteen participants with fibromyalgia were included in this study. EEG recordings were done during resting state and hypnosis conditions. After both conditions, levels of pain, comfort, absorption, and dissociation were assessed using a numerical rating scale. Time perception was collected via an open-ended question. The study was prospectively registered in the ClinicalTrials.gov public registry (NCT04263324). **Results**: Neural oscillations showed increased theta power during hypnosis in the left parietal and occipital electrodes, increased beta power in the frontal and left temporal electrodes, and increased slow-gamma power in the frontal and left parietal electrodes. Functional connectivity using pairwise-phase consistency measures showed decreased connectivity in the frontal electrodes during hypnosis. Graph-based measures, the node strength, and the cluster coefficient were lower in frontal electrodes in the slow-gamma bands during hypnosis compared to resting state. Key findings indicate significant changes in neural oscillations and brain functional connectivity, suggesting potential electrophysiological markers of hypnosis in this patient population.

## 1. Introduction

Hypnosis is a modified state of consciousness characterized by focused attention, absorption (in the experience), dissociation (separation of mental processes, bodily awareness, and perceptions) [1], and the suspension of awareness of the environment, which, when combined with specific suggestions, produces a rich phenomenology [2,3,4]. Currently, electroencephalographic (EEG) studies have yielded no consensus on a neurophysiological signature of hypnosis [5]. Some studies have reported a general increase in alpha (α) activity [6], some reported decreased α activity [5,7,8], while others found no activation of α bands during hypnosis [6]. Another study found that, compared to low hypnotizable participants, highly hypnotizable participants had significantly higher theta (*θ*) activity in the occipital, central, and frontal regions both at baseline and during hypnosis [9,10]. However, these findings have not been consistently replicated [11]. A study on highly hypnotizable participants recently indicated that *θ* activity might not be a reliable EEG marker of hypnotic suggestibility (i.e., hypnotizability; heightened responsiveness to hypnotic suggestions) [11]. Although Jensen and collaborators [12] hypothesized that *θ* activity plays a central role in hypnosis and suggestibility, this assumption has yet to be demonstrated, as findings seem inconsistent. Neuroimaging studies show that hypnosis acts upon three brain networks: the default mode network (autoreferential processing, including the medial prefrontal cortex, mPFC), the salience network (including the anterior cingulate cortex (ACC) and insula), and the executive control network (including the dorsolateral prefrontal cortex, dlPFC) [13]. It was proposed that the modified activation and connectivity of these three intrinsic brain networks account for reduced mind wandering and self-referential processing, as well as an altered sense of agency (i.e., to have control and influence in life), which are the phenomenological characteristics of hypnosis [13]. However, further analyses showed no consistent brain pattern of activation/deactivation except for the activation of the lingual gyrus, a brain area responsible for advanced visual processing and mental imagery [13].

In addition to studying hypnosis processes, research on hypnosis has also focused on examining the clinical relevance of this peculiar state of consciousness. Hypnosis is indeed also known for its benefits in chronic pain management [14]. Studies highlight that hypnosis decreases pain, as well as numerous comorbidities, such as anxiety, depression, and sleep disorders, enhancing the global quality of life of patients suffering from this worldwide burden [15]. Only a few studies have investigated brain activity during hypnosis in chronic pain, and most of them relied on functional magnetic resonance imaging (fMRI) [16]. Consistent with the behavioral benefits seen after hypnosis, neuroimaging studies have revealed that hypnosis primarily affects regions within the corticolimbic system involved in the emotional and motivational processing of pain, and to a lesser degree, the sensory components of pain [16]. When comparing hypnotic analgesia to an ordinary awake state, two studies using EEG showed reduced somatosensory event-related potentials during a cold pressing task [17] and reduced laser-evoked potentials [18] during hypnosis in patients with chronic pain of various etiologies. This highlights the fact that hypnosis is a relevant tool to reduce pain both in experimental and clinical settings.

Fibromyalgia is an idiopathic syndrome portrayed by chronic musculoskeletal pain, sleep disorders, fatigue, mood disorders (anxiety, depression, etc.), and poor quality of life [19]. More specifically, diagnosis of fibromyalgia requires the presence of pain in at least four body regions (among head, neck, arms, chest, abdomen, upper and lower back, spine, and legs); associated symptoms, such as sleep disorders, cognitive dysfunction, and somatic symptoms; and the presence of these symptoms for at least 3 months [20]. According to a recent review, 1 to 9 percent of the general population is affected by fibromyalgia, with a higher incidence in women [21]. Few studies have investigated resting-state EEG in patients with fibromyalgia. Hargrove et al. [22] found decreased *δ*, *θ*, and α absolute power and increased *β* relative power in patients with fibromyalgia compared to healthy controls. Moreover, coherence was low compared to healthy controls. These results were observed in frontal regions. Gonzalez-Roldan et al. [23] went on to confirm part of Hargrove et al.’s [22] results, as they observed decreased *δ* power and increased *β*2 and *β*3 power. Source analyses showed that reduced *δ* power was pertained to the right insula and temporal cortices; whereas, increased *β* power was located in the right frontal, midcingulate, and motor regions in patients with fibromyalgia. These regions are all implicated in the sensory and affective processing of pain. Reversely, these authors [23] found increased coherence at *δ*, *θ*, α, and *β* bands over the left hemisphere compared to healthy controls. On a more clinical note, results showed that pain duration was significantly correlated with reduced *δ* power in the insula, while anxiety and depression scores were associated with *β*3 power in the frontomedial regions. Moreover, anxiety scores were also correlated with *β*3 power in the midcingulate areas. This pattern of activation was interpreted as an increase in excitatory processes in the brain of patients with fibromyalgia during resting state [23]. More recently, a study found increased coherence between the right ACC and the left dlPFC in the *β*3 band, as well as enhanced interhemispheric connectivity between the insula and the dlPFC and the insular areas still in the *β*3 band [24]. These regions are implicated in pain processing and are part of the salience and executive control networks. On the other hand, Martin-Brufau [25] found that, except for the *δ* band, patients with fibromyalgia display lower power in all frequencies during resting state compared to healthy controls.

Studies investigating hypnosis’s benefits for fibromyalgia management show promising results regarding pain reduction, reduction in psychological distress, and improvement of sleep, even though these results are of low-quality evidence [26,27,28]. Therefore, hypnosis appears to be a suitable approach for the management of patients with fibromyalgia, but its mechanisms of action remain poorly understood, and no EEG study has investigated the neurophysiology of hypnosis in patients with fibromyalgia in the absence of painful stimulation.

Building on these considerations, the present study aims at assessing whether hypnosis leads to a modification in electrophysiology, as compared to an ordinary awake resting state in patients with fibromyalgia. Since there is no clear evidence of a consistent electrophysiological signature of hypnosis, and since this study is the first to assess electrophysiology in patients with fibromyalgia, no a priori hypothesis was advanced.

## 2. Materials and Methods

### 2.1. Population

The study started on 18 March 2019 and ended on 27 January 2020, when we included the last participant. Participants were eligible for inclusion if they were at least 18 years old, fluent in French, had a medical diagnosis of fibromyalgia, and had stable pharmacological treatment (i.e., during the last 4 months). Exclusion criteria were the presence of psychiatric disorders (schizophrenia, psychosis, borderline with prolonged dissociation episodes), neurological disorders (epilepsy), previous brain injury, cancer, drug addiction, and alcoholism. All participants gave written informed consent to participate in the study.

### 2.2. Experimental Procedure

The study was part of a larger study assessing the impact of learning self-hypnosis/self-compassion on the brain functioning of patients with fibromyalgia using fMRI and EEG. Due to the COVID-19 pandemic, the study had to be stopped, but we still decided to analyze the data that had been acquired (i.e., resting state and hypnosis with fMRI and EEG). This study only addresses the EEG data. The study was approved by the Ethics Committee of the Medical School of the University of Liège, Belgium (internal reference number: 2017/291, date of acceptance: 8 January 2018, President at the time of the study: Vincent Seutin) and prospectively registered in the ClinicalTrials.gov public registry (NCT04263324).

Participants were invited to experience two hypnotic conditions on the same day. One was carried out in a fMRI scanner (fMRI session), while the other was in a quiet experimental (nonshielded) room with an EEG cap on for recording (EEG session). During the EEG session, participants sat on a comfortable chair and were informed to close their eyes throughout the experiment and to stay awake and still. Both sessions were conducted in the same way: they started with a resting state (REST, mean: 12.2, SD: 0.8 min) condition, and then, one of the experimenters (AV) induced hypnosis (i.e., hypnosis (HYP) condition, mean 15.8, SD: 1.9 min). High-density EEG (saline-based 256 electrodes with 500 Hz sampling rate, EGI, Electric Geodesics, Eugene, OR, USA) and body physiological sensors (auxiliary input box of EGI, Electric Geodesics, Eugene, OR USA) consisting of two electrodes on the chest for electrocardiogram were recorded during both conditions. The recordings were started after hypnotic induction in the HYP condition.

The hypnosis exercises started with a 3 to 8 min induction, consisting of progressive eye fixation, body scan, and muscle relaxation; the time varied based on the experimenter’s (AV) observations of the participant’s behavior and needs. Participants were then prompted to relive a pleasant autobiographical memory. Permissive and indirect suggestions were used to enhance the hypnotic state, with continuous cues provided to maintain it.

**Figure 1:**
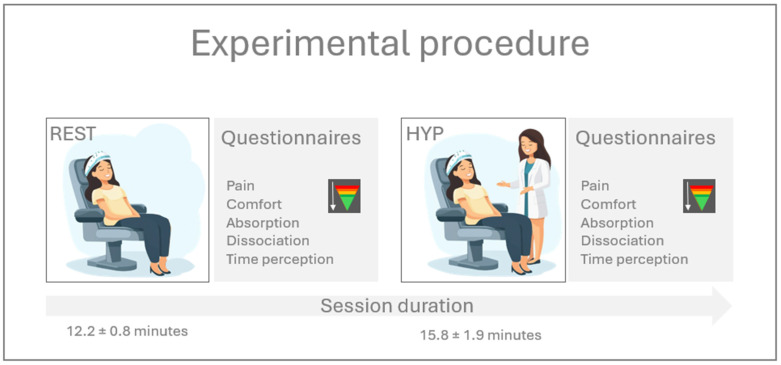
Experimental procedure. REST: resting state; HYP: hypnosis.

After both conditions (REST and HYP), numerical rating scales were used to assess the levels of pain (0 = no pain at all, 10 = worst imaginable pain), comfort (0 = no comfort at all, 10 = complete comfort), absorption (0 = not absorbed at all, 10 = fully absorbed in the experience), and dissociation (0 = to be in reality, in this room, 10 = to fully be disconnected from the here-and-now reality). Time perception was also gathered with an open-ended question, where participants were asked to estimate the duration of conditions (Figure 1). Questionnaires related to absorption, dissociation, and time perception were shown to be a good indicator to characterize the subjective experience of hypnosis in healthy volunteers [29]. In addition, these questionnaires have been used in several peer-reviewed studies related to nonordinary states of consciousness [30,31,32,33]. All recordings were carried out in the morning.

### 2.3. Analyses

#### 2.3.1. Behavioral Data

Descriptive statistics were first conducted. The normality of quantitative variables was examined graphically with histograms and quantile–quantile plots and statistically by performing a Shapiro–Wilk normality test. Quantitative variables were reported as means and standard deviations (SD) or median and interquartile range (IQR), depending on data distribution. Qualitative variables were expressed as counts and percentages. Univariate analyses were performed to compare scores between the two conditions (i.e., REST vs. HYP) using a paired Student’s *t*-test for each variable. Results were considered statistically significant at the 5% critical level (*p* < 0.05). All statistical analyses of behavioral data were performed using the statistical data processing software Jamovi 2.2.5 [33].

#### 2.3.2. Electrophysiological Data and Preprocessing

The EEG data were preprocessed using the MILE preprocessing pipeline [34] using the EEGLAB toolbox version v2021.1 [35]. The EEG data were first bandpass filtered between 1 and 95 Hz. The notch filter was applied at the 50 Hz line frequency. Channels with low-quality signal (bad channels) were identified with power spectrum density (PSD). A channel for which the PSD was above or below three times the standard deviation in at least 30 percent of the frequency bins was marked as a bad channel. Artifacts such as movement artifacts were identified and rejected automatically based on temporal and spectral features [34]. Independent component analysis (ICA) decomposition was carried out using the runica algorithm [36]. After the removal of muscle, eye, heart, and line-noise-related ICA components using an automated independent component classifier toolkit [37], bad channels were interpolated with the spherical interpolation method, resulting in 172 channels. Hence, the number of electrodes across all subjects for further analysis was consistent with 172 channels. The bad segments in the data not identified by the preprocessing steps were detected and removed by visual inspection. The preprocessing pipeline used in the study has been used in multiple studies [34,38]. The first 10 min of free-artifact data (600 epochs each of size one second) each, both for the REST and HYP conditions, were considered for further analysis. All the analysis was carried out at the sensor/scalp level. The two-tailed Wilcoxon signed-rank test [39] was applied for the statistical comparisons of EEG measures, such as PSD, coherence, and graph-based measures between the different conditions, at a *p* < 0.05 significance threshold. The data were analyzed for different canonical frequency bands, namely delta *δ*: 1–4 Hz, theta *θ*: 5–8 Hz, alpha α: 9–13 Hz, beta1 *β*1: 14–17 Hz, beta2 *β*2: 18–23 Hz, beta3 *β*3: 24–30 Hz, slow gamma s*γ*: 31–45 Hz, and fast gamma f*γ*: 55–95 Hz. The analysis is considered until 95 Hz, which is generally considered the fast gamma band due to the reported literature on the role of f*γ* oscillations in cognition [40] and emotion [41]. This was also supported by the reliable preprocessing method employed in the study, which reduces the noise related to muscle artifacts in f*γ* bands [34]. Because of high-density recording, a sufficient number of electrodes were present in each lobe, and hence, the analyses were carried out across hemisphere and lobe wise (frontal, central, temporal, parietal, occipital, anterior midline, and posterior midline).

#### 2.3.3. Power Spectrum Estimation

The preprocessed data were downsampled to 250 Hz, and the power spectrum was estimated using a multi-taper method with a single Slepian taper, as provided in the Chronux toolbox version 2.12 v03 [42]. The power spectrum was estimated with a frequency resolution of 1 Hz, and the range of 1–95 Hz was considered for testing statistical significance using the Wilcoxon rank-sum test. Spectrum analysis was carried out at the whole brain level and subsequently across different lobes. Absolute total band power was estimated for all the channels in each of the considered canonical frequency bands, as mentioned in the electrophysiological data and preprocessing section. False discovery rate (FDR) correction was not applied due to small sample size.

#### 2.3.4. Functional Connectivity and Graph Theory Measure Estimation

Functional connectivity (FC) was carried out using the Fieldtrip toolbox version 2024-0118 [43]. Pairwise-phase consistency (PPC) [44] addresses the limitations of coherence, particularly by focusing on the phase relationships between signals, which reduces the influence of common sources and spurious correlations and is also unbiased with the number of epochs. PPC’s focus on nonzero lag phase relationships allow it to detect more biologically meaningful connections and the degree of functional connectivity using phase consistency between each pair of electrodes using nonzero lag phase relationships; it was estimated over 1–95 Hz in the frequency range, with a frequency spacing of 1 Hz, resulting in 95 connectivity matrices for a given condition and participant [43,45]. Graph theory measures the node strength, measuring the depth of the nodes ties, and the clustering coefficient, measuring the degree to which nodes in a graph tend to cluster locally [6,46,47]; they were estimated using the connectivity matrices and were subjected to Wilcoxon signed-rank test.

## 3. Results

### 3.1. Population

Twenty-two participants marked their interest in participating in the study. Out of those participants, 13 were included in the current study. One participant was excluded because of an additional diagnosis of leukemia known after the inclusion procedure, one did not come on the experimental day, two canceled their participation, one had frizzy hair preventing the use of the EEG device, and one dropped out because the fMRI sounds led to them re-experiencing traumatic events. Behavioral data were missing for one participant, due to technical issues, EEG data were missing for two participants. The mean age of the final sample was 48.2 (SD: 9.57) years, all were women, nine women had an intermediate educational level (12 years of school), and four had a high educational level (≥15 years of school). Mean pain duration was 11.1 (SD: 9.70) years. All patients reported having remained awake throughout the duration of the experiment.

### 3.2. Behavior Data

All participants confirmed that they experienced a modified state of consciousness during the HYP condition. Dissociation significantly increased (*t*_(12)_ = −3.180, *p* = 0.008) during the HYP condition, as compared to the REST condition. No other comparisons reached statistical significance (Table 1).

### 3.3. Electrophysiology Data

#### 3.3.1. Spectrum Analysis

**Figure 2:**
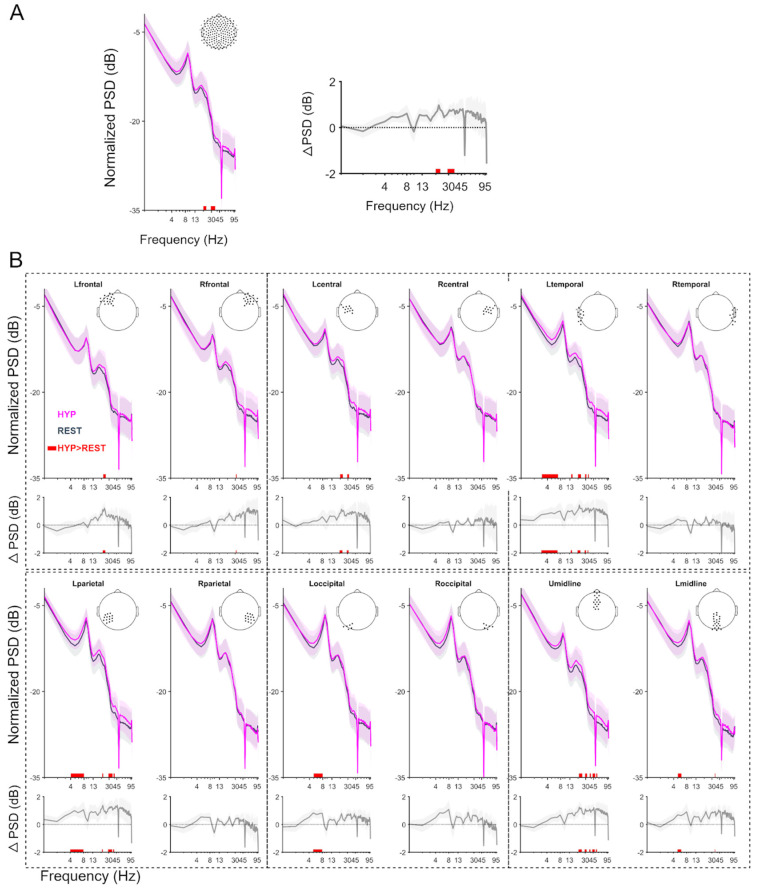
Power spectrum plots during resting state (REST) and hypnosis (HYP) across subjects and ΔPSD is the difference in PSD between HYP and REST. (**A**) across the whole brain (left), the mean difference of PSD (right) and (**B**) for different groups of electrodes. The 2nd and 4th rows show the difference in power between the hypnosis and the resting state conditions. The red shaded patch depicts the significant (*p* < 0.05 without FDR correction) increase in HYP power compared to REST. The inset topoplots represent the group of electrodes considered for respective lobe-wise representation. L: left, R: right, U: upper, L: Lower, Hz: hertz, PSD: power spectral density. Solid lines represent the group mean and the shaded regions represent the standard error of the mean (SEM).

Figure 2 depicts significant differences between HYP and REST regarding spectrum analysis. PSD at the whole brain level obtained by averaging all the electrodes showed increased oscillations in *β* and s*γ* bands during HYP, as shown in Figure 2A. To study the different brain region contribution, this analysis was carried out in different regions or lobes, which are given in Figure 2B. A significant increase in *θ* activity was observed in the left temporal, parietal, and occipital electrodes during HYP. An increase in *β* band activity during hypnosis was observed in the left frontal, central, temporal, and parietal electrodes and upper midline electrodes. s*γ* activity was also increased during HYP in the left temporal and upper midline electrodes, as compared to the REST condition. No significant changes were observed in the α band. However, no decrease in activity in any of the studied frequency bands was observed during HYP compared to REST.

**Figure 3:**
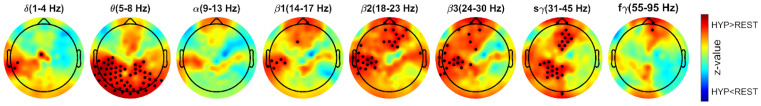
Change in absolute band power during the HYP condition compared to the REST condition across different frequency bands. The highlighted electrode represents significant differences (*p* < 0.05 without false discovery rate correction). Color bar indicates the z-values, REST: resting state, HYP: hypnosis.

The difference in absolute band power between HYP and REST is illustrated in Figure 3. Total band power in the *δ* band during HYP was higher in the left temporal electrode. *θ* band power is increased significantly in the posterior electrodes during HYP, as compared to the resting state. No significant changes were observed in the α band. The *β* band power increased in the left and mid frontal and parietal electrodes during HYP. The band power in the s*γ* band significantly increased in the mid-frontal and left posterior electrodes during HYP. One electrode in the mid-frontal region showed increased f*γ* activity during HYP, as compared to REST.

#### 3.3.2. Functional Connectivity and Graph Based Measure Analysis

**Figure 4:**
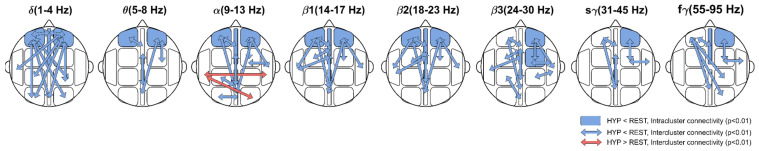
Pairwise-phase consistency (PPC) during hypnosis (HYP) condition compared to resting state (REST) condition across different frequency bands. The highlighted regions and arrows represent the significant connectivity (*p* < 0.01 without FDR correction). Different sets of electrodes are grouped lobe wise, as given in the inset of Figure 2B.

**Figure 5:**
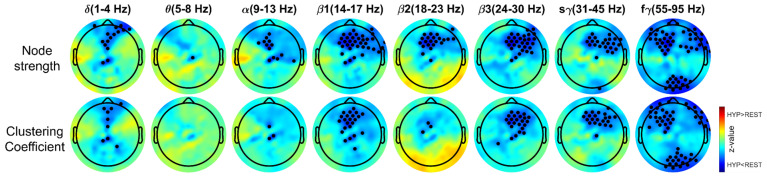
Change in graph theory measures clustering coefficient and node strength during HYP compared to REST across different frequency bands. The highlighted electrode represents significant differences (*p* < 0.05 without FDR correction). The color bar represents z-value. REST: resting state, HYP: hypnosis.

Functional connectivity estimated using pairwise-phase consistency is shown in Figure 4. A significant decrease in anterior region electrode connectivity was observed during HYP across all bands. The anterior to posterior connectivity decreased during HYP, as compared to REST. Decreased anterior-to-posterior midline connectivity was observed in all bands except the *δ* band. In the *β* band, decreased connectivity was observed between the temporal and frontal electrodes. Increased connections were observed laterally between the left temporal, right temporal, and occipital electrodes in α band. Decreased connectivity was observed between the anterior left and posterior right electrodes in f*γ* band. The node strength and clustering coefficient were estimated to characterize functional connectivity (Figure 5). Node strength significantly decreased during HYP in the mid-frontal region electrodes in all bands except the *θ* band and right frontal electrodes in the *β* and *γ* bands. Similar observations were made in the clustering coefficient measure except in the α and *β*2 bands.

## 4. Discussion

The current study aimed to determine whether hypnosis causes changes in neurophysiology compared to ordinary resting state in patients with fibromyalgia. Given the lack of clear evidence for a consistent change in neural oscillations during hypnosis and the fact that this was the first study to examine hypnosis-related neurophysiology in patients with fibromyalgia, no specific hypothesis was advanced.

Behavioral results indicate a significant increase in dissociation during the HYP condition, as compared to the REST condition. Dissociation has long been linked to hypnosis and, within this context, does not appear to be pathological, as it is reversible [48,49]. Several studies conducted with healthy volunteers have highlighted increased dissociation, as self-assessed by participants during a hypnotic experience [6,29,38]. Moreover, pain and comfort perceptions did not significantly change in the HYP condition. This is not in line with previous studies. Indeed, a decrease in pain sensation was reported in patients with fibromyalgia and patients suffering from chronic back pain during one hypnotic session, including analgesic suggestions combined with emotional-weighted network activation (e.g., prefrontal cortex, insula and subcortical brain areas), compared to a control resting state condition, as evidence by positron emission tomography [50,51]. This discrepancy could result from the difference between hypnotic protocols, since we did not specifically use hypno-analgesia suggestions in our study. To note, even though not significant, results indicate a decrease in pain perception and an increase in comfort. Those results might, thus, also reflect a lack of statistical power, as other studies using non-pain-related suggestions showed decreased pain perception in patients with fibromyalgia [52,53,54,55,56,57].

Regarding the results estimated from EEG, the findings of the present study indicate that the HYP condition lead to increased *θ* power in the posterior and left temporal regions, *β* power in the antero-posterior and midline areas, s*γ* power in the temporal and midline regions, and *δ* power in the temporal cortex compared to the REST condition. These findings pertained to the left hemisphere only. Pairwise-phase consistency decreased in the anterior regions for all bands and in the antero-posterior regions in *δ* and f*γ* bands. Conversely, pairwise-phase consistency increased between the left and right temporal regions’ *θ* bands.

Amongst the neural oscillations, the one most linked to hypnosis and hypnotic suggestibility is the *θ* band, as it was shown that highly hypnotizable people have more *θ* activation during both resting state and hypnosis [12,58]. The current study results are in line with these studies, showing increased *θ* power in the posterior regions. Increased *γ* during hypnosis has also been reported, particularly for participants with a high hypnotic suggestibility (see [59] for a review). The increased s*γ* power in the temporal and midline regions during the HYP condition may be related to the slow waves, such as *θ*, potentially controlling the fast waves, such as *γ*, through phase-linked mechanisms, and that could influence hypnotic responsiveness [12]. However, this theory has to be studied further to be confirmed. Indeed, other findings are inconsistent with the assumption that *θ* plays a central role in hypnosis, since results showed no activation of the *θ* band [11]. Similar to the present findings, Lipari et al. [60] found increases in *δ* and *β*1 bands in the parietal cortex, while also observing decreases in the *θ*, α, *β*2, and *β*3 bands in the left visual cortex, supramarginal gyrus, anterior cingulate cortex, and anterior prefrontal cortex. Others also found contrasting results with decreased α and increased *δ* power in the frontoparietal regions during hypnosis when compared to normal wakefulness [8,61]. The left lateralization in the significant electrodes across the *θ*, *β*, and s*γ* bands may be confounded due to the continuous auditory inputs during the HYP condition, leading to the activation of the left temporal region of the brain, which require further studies to isolate the effects due to hypnosis.

Regarding pairwise-phase consistency as a connectivity measure, previous studies reported decreased coherence connectivity for *δ*, α, *β*, and *γ* bands in posterior regions [62], as well as decreased global connectivity in highly compared to slightly hypnotizable participants [63]. Panda et al. [6] found decreased α and *β*2 bands in phase-lag index connectivity in the frontal-midline and midline regions and increased *δ* band connectivity in the anterior and antero-posterior regions during hypnosis compared to resting state in highly hypnotizable healthy participants. Those results are in line with the present findings, as well as with the more general findings, on connectivity during hypnosis [59]. Also, in the current study, *θ* band connectivity in the frontal region and right frontal-to-posterior midline was decreased during the HYP condition compared to the REST condition, which could be due to the continuous tonic pain in fibromyalgia patients [64] and may also be confounded by the continuous auditory inputs during the HYP condition.

As previous studies relied on positron emission tomography, fMRI, or event-related potentials to assess the effect of hypnosis (with or without analgesia suggestions) on pain perception (with or without painful stimulation) of patients with fibromyalgia [15], the interpretation of our results are extrapolative and, therefore, hypothetical. In short, these few studies showed that hypnosis targets brain regions involved in the corticolimbic system, hypothesizing that hypnosis alters the emotional/motivational processing of pain, in addition to the sensory component of pain [16]. Moreover, and similar to our findings, those studies showed that hypnosis impacts the activation and coherence in the midline, frontal, and antero-posterior regions, known to be related to self-awareness [65]. Activation and connectivity within and between these regions are also known to be disrupted in patients with fibromyalgia [66,67]. A recent fMRI study indeed showed that patients experiencing pain during scanning had increased connectivity between the midline and the insula compared to patients who did not feel any pain during scanning [68]. The patients in the present study showed reduced connectivity at the frontal level and increased s*γ* and *δ* band power in the midline during the HYP condition, reflecting a modified state of consciousness and possibly altered auto-referential processing. This could then lead to reduced pain perception. Although not significant, pain decreased, and comfort increased during the HYP condition outlining a trend in favor of our hypothesis. Again, this is speculative, as the current design, with its own limitations, prevents us from drawing firm conclusions. Nevertheless, this study being the first of its kind, paves the way for more robust settings.

This study has some limitations. First, the relatively small sample size prevents us from making any generalizations and may have prevented us from evidencing some statistically significant differences. Second, the analyses were not carried out blindly, which might have induced a detection or confirmation bias. Third, given the nature of hypnosis, the experimenter was talking during the EEG recordings; it is, thus, possible that the results reflect auditory processing, since any verbal input (hypnotic or not) increases the *θ* band power [69]. Fourth, the order of the procedure was not randomized between participants, which could have led to an order effect. Fifth, hypnotic suggestibility was not assessed. Given that most of the neuroscientific literature seeks differences between high and low hypnotizable participants, it would have rendered the discussion less hypothetical. Finally, this study did not include healthy volunteers as a control group.

## 5. Conclusions

This is the first study assessing hypnosis-related resting state electrophysiology in patients with fibromyalgia and, by extension, chronic pain. Behavioral results replicate an increase in dissociation, as self-reported by patients, similarly to what was previously observed in healthy volunteers. EEG results indicate a significant modification of brain electrical activity, both regarding power and coherence of the brain during hypnosis compared to an ordinary resting state. Specifically, we observed the left lateralization of modulations with increased *θ* power in posterior regions, *β* power in antero-posterior and midline areas, s*γ* power in temporal and midline regions, as well as *δ* power in the temporal cortex during hypnosis. Our findings are consistent with evidence that hypnosis influences activation and coherence in the midline, frontal, and antero-posterior brain regions, which are associated with modified self-awareness and altered auto-referential processing during hypnosis. However, these results come from studies utilizing neuroimaging techniques other than EEG and different hypnotic protocols, even though they involved fibromyalgia patients. This limits the interpretation of our current findings. While the findings foster our understanding of hypnosis, more robust studies involving patients suffering from chronic pain are needed to better understand how hypnosis works in this population.

## Figures and Tables

**Table 1:** Means, standard deviations, and *p*-values of each outcome in both conditions, i.e., resting state and hypnosis.

	REST	HYP	
Outcomes			*p*-Value
Pain, NRS, 0–10	4.23 (2.95)	3.23 (3.03)	0.235
Comfort, NRS, 0–10	8.23 (1.48)	8.46 (1.20)	0.570
Absorption, NRS, 0–10	7.31 (2.50)	7.23 (2.09)	0.746
Dissociation, NRS, 0–10	4.46 (3.23)	7.23 (2.09)	0.008
Time perception, open question	10.5 (7.26)	12.3 (6.33)	0.398

NRS = numerical rating scale, REST: resting state, HYP: hypnosis.

## Data Availability

The data are available on request to the corresponding authors due to ethical reasons.
